# Assessment of Clinical Information Quality in Digital Health Technologies: International eDelphi Study

**DOI:** 10.2196/41889

**Published:** 2022-12-06

**Authors:** Kayode Philip Fadahunsi, Petra A Wark, Nikolaos Mastellos, Ana Luisa Neves, Joseph Gallagher, Azeem Majeed, Andrew Webster, Anthony Smith, Brian Choo-Kang, Catherine Leon, Christopher Edwards, Conor O'Shea, Elizabeth Heitz, Olamide Valentine Kayode, Makeba Nash, Martin Kowalski, Mateen Jiwani, Michael Edmund O'Callaghan, Nabil Zary, Nicola Henderson, Niels H Chavannes, Rok Čivljak, Olubunmi Abiola Olubiyi, Piyush Mahapatra, Rishi Nannan Panday, Sunday O Oriji, Tatiana Erlikh Fox, Victoria Faint, Josip Car

**Affiliations:** 1 Department of Primary Care and Public Health Imperial College London London United Kingdom; 2 Centre for Intelligent Healthcare Institute for Health and Wellbeing Coventry University Coventry United Kingdom; 3 gHealth Research Group School of Medicine University College Dublin Dublin Ireland; 4 Leeds Teaching Hospital National Health Service Trust Leeds United Kingdom; 5 Health Board Hywel Dda University Wales United Kingdom; 6 Glasgow Royal Infirmary National Health Service Greater Glasgow and Clyde Glasgow United Kingdom; 7 Oxford University National Health Service Foundation Trusts Oxford United Kingdom; 8 Leeds Children’s Hospital Leeds United Kingdom; 9 Wheaton Hall Medical Practice Drogheda, Co Louth Ireland; 10 Croydon Health Services Croydon United Kingdom; 11 Department of Paediatrics and Child Health Evercare Hospital Lekki Lagos Nigeria; 12 Department of Ophthalmology University Eye Hospital Tübingen Tübingen Germany; 13 School of Medicine University of Limerick Limerick Ireland; 14 Institute for Excellence in Health Professions Education Mohammed Bin Rashid University of Medicine and Health Sciences Dubai United Arab Emirates; 15 National Health Service Forth Valley Scotland United Kingdom; 16 National eHealth Living Lab Department of Public Health and Primary Care Leiden University Medical Center Leiden Netherlands; 17 University Hospital for Infectious Diseases Zagreb Croatia; 18 University of Zagreb School of Medicine Zagreb Croatia; 19 Medical Research Council Unit The Gambia London School of Hygiene & Tropical Medicine Fajara Gambia; 20 West Hertfordshire Teaching Hospitals National Health Service Trust Watford United Kingdom; 21 Acute Medicine Section Department of Internal Medicine Amsterdam University Medical Centers, VU Medical Center Location Amsterdam Netherlands; 22 Department of Mental Health Nnewi Campus Nnamdi Azikiwe University Teaching Hospital Nnewi Nigeria; 23 Centre for Population Health Sciences Lee Kong Chian Medicine Nanyang Technological University Singapore Singapore

**Keywords:** information quality, digital health technology, patient safety, perspective, digital health technologies, DHT, thematic analysis, clarity, understandable, understandability, readability, searchability, security, decision support system, framework development, framework

## Abstract

**Background:**

Digital health technologies (DHTs), such as electronic health records and prescribing systems, are transforming health care delivery around the world. The quality of information in DHTs is key to the quality and safety of care. We developed a novel clinical information quality (CLIQ) framework to assess the quality of clinical information in DHTs.

**Objective:**

This study explored clinicians’ perspectives on the relevance, definition, and assessment of information quality dimensions in the CLIQ framework.

**Methods:**

We used a systematic and iterative eDelphi approach to engage clinicians who had information governance roles or personal interest in information governance; the clinicians were recruited through purposive and snowball sampling techniques. Data were collected using semistructured online questionnaires until consensus was reached on the information quality dimensions in the CLIQ framework. Responses on the relevance of the dimensions were summarized to inform decisions on retention of the dimensions according to prespecified rules. Thematic analysis of the free-text responses was used to revise definitions and the assessment of dimensions.

**Results:**

Thirty-five clinicians from 10 countries participated in the study, which was concluded after the second round. Consensus was reached on all dimensions and categories in the CLIQ framework: informativeness (accuracy, completeness, interpretability, plausibility, provenance, and relevance), availability (accessibility, portability, security, and timeliness), and usability (conformance, consistency, and maintainability). A new dimension, searchability, was introduced in the availability category to account for the ease of finding needed information in the DHTs. Certain dimensions were renamed, and some definitions were rephrased to improve clarity.

**Conclusions:**

The CLIQ framework reached a high expert consensus and clarity of language relating to the information quality dimensions. The framework can be used by health care managers and institutions as a pragmatic tool for identifying and forestalling information quality problems that could compromise patient safety and quality of care.

**International Registered Report Identifier (IRRID):**

RR2-10.1136/bmjopen-2021-057430

## Introduction

Digital health technologies (DHTs), such as electronic health records, electronic prescribing systems, and clinical decision support systems, have transformed health care delivery around the world [1]. However, the quality of information obtained from DHTs varies and can compromise quality and safety of care [2-4]. Several incidents of delayed, missing, partial, or wrong information in DHTs have been documented, resulting in adverse patient outcomes, including death [3-5]. To reduce the risk of such incidents, we need a pragmatic approach to assessing the quality of clinical information in DHTs. The importance of such an information quality assessment tool continues to grow with increasing automation and use of artificial intelligence (AI) in health care, as human checks are reduced and clinical information feeds into AI tools and algorithms [6].

A systematic review of the literature identified existing frameworks and dimensions that are relevant to assessing clinical information in DHTs [7]. However, the review found that the existing frameworks did not provide assessment tools for clinical practice [7]. In addition, most of the existing frameworks were developed without input from clinicians who use clinical information from DHTs [7]. Drawing on the review’s findings, we developed a clinical information quality (CLIQ) framework as a pragmatic approach to assessing the quality of clinical information in DHTs. The CLIQ framework defined 13 dimensions relevant to the quality of clinical information in DHTs and was accompanied by a questionnaire for assessing information quality. The current study explored clinicians’ perspectives on the relevance, definition, and assessment of information quality dimensions in the CLIQ framework (Textbox 1 shows the original dimensions in the CLIQ framework).

**Information quality dimensions in the original CLIQ framework.**

- Informativeness (accuracy, completeness, interpretability, plausibility, provenance, and relevance)
- Availability (accessibility, portability, security, and timeliness)
- Usability (conformance, consistency, and maintainability)

## Methods

### Study Design

In this study, the eDelphi method was used to obtain direct input from clinicians to contextualize the CLIQ framework to the needs of the information users. This method uses a systematic process for engaging and integrating the opinions of multiple experts to reach consensus [8,9]. Thus, the eDelphi method was suitable for this study, which sought to obtain the consensus of clinicians from different countries on the information quality dimensions that are relevant to assessing clinical information in DHTs. In addition, the asynchronous approach gave the panelists an opportunity for equal participation, in contrast to physical meetings, which are usually dominated by a few outspoken participants [10]. The iterative process of the eDelphi method enabled the participants to provide feedback and reconsider their opinions based on collective responses [11]. The flexibility of the eDelphi method allowed collection of quantitative and qualitative data, which were useful in addressing the research question.

### Ethics Approval

The protocol of this study was published to promote transparency [12]. Ethics approval was obtained for the study from the Imperial College Research Ethics Committee (20IC6396).

### Steering Committee

This eDelphi study was coordinated by a steering committee comprising health care researchers and clinicians with interest in digital health. The committee developed the original CLIQ framework [7] and the accompanying questionnaire from which the initial items of the eDelphi study were generated. The committee recruited the participants to the study and made decisions regarding retention, removal, or redefinition of information quality dimensions based on the input of the participants according to prespecified decision and stoppage rules.

### Decision and Stoppage Rules

The decision and stoppage rules on consensus were predefined to prevent bias during analysis [11]. An information quality dimension was considered relevant and was retained in the final framework when at least 70% of the participants, in any round of the survey, chose the options “strongly relevant” or “somewhat relevant.” The choice of 70% as a cutoff was a pragmatic choice based on the literature, as most Delphi studies use 60% agreement or higher as a threshold for consensus [10]. The study was planned to be concluded whenever consensus was reached on at least 80% of the dimensions or at the end of the third round, irrespective of the level of consensus [11].

### Participant Recruitment

Clinicians with information governance roles or interest were invited to participate in the eDelphi panel based on the following eligibility criteria [12]: (1) prior or current experience of using DHTs in patient care, (2) information governance role or personal interest in information governance, and (3) willingness to participate in a multiple-round eDelphi study (up to 3 rounds).

The heterogeneity of the participants provided a wide range of perspectives and increased the study’s external validity. The recruitment of the participants included both purposive and snowball sampling. Clinicians with information governance roles (eg, chief clinical information officer, chief nursing information officer, or Caldicott guardian) were targeted, as they have both DHT user experience and information governance expertise. However, participation was not restricted to these roles, as they do not exist in many low- and middle-income countries. Therefore, participants with interest in information governance without any formal information governance role were also recruited, such as clinicians who have published papers relating to information governance.

The steering committee members nominated clinicians from within and beyond their professional networks. Each eligible clinician was invited by an introductory email containing a link to the survey; the email also encouraged them to share the invitation with other eligible clinicians. Two reminders were sent at least 2 weeks apart to encourage participation [8]. Thirty-five clinicians from 10 countries participated in the study, including doctors, nurses, pharmacists, and other health care professionals.

### Survey Content and Administration

The initial survey (Multimedia Appendix 1) was generated from the CLIQ framework [7] and the accompanying assessment questionnaire. The accompanying assessment questionnaire was developed by the steering committee based on the findings of a systematic review of information quality frameworks [7] and further evidence from literature. The survey was administered in English.

The introductory section of the survey provided brief information about the study, a link to the participant information sheet, and the electronic consent form. Demographic data were collected from participants who gave informed consent, and only these participants were shown the remainder of the survey.

The second section of the survey consisted of questions relating to the CLIQ framework. The first part of this section included 5-point Likert scale questions on the relevance of the dimensions in the CLIQ framework to quality and safety of care. The Likert scale captured a range of options (strongly relevant, somewhat relevant, neither relevant nor irrelevant, somewhat irrelevant, and strongly irrelevant) that represent categories people naturally create and thus did not require a heavy cognitive load. The second part comprised multiple-choice and free-text questions on the definition, assessment, and categories of the dimensions in the CLIQ framework. Finally, the email addresses of participants were collected for feedback purposes and as a contact method for the next round of the survey. The survey was set up using Qualtrics software (Qualtrics) and piloted by the steering committee members before its administration. The study was conducted between June 2021 and March 2022.

### Data Analysis

The data on the relevance of the dimensions were summarized using descriptive statistics and used to inform decisions on retention of dimensions and termination of the study. The data were also used to provide feedback to the participants during the second round of the survey. The free-text suggestions were analyzed using a reflexive thematic analysis approach, which allowed the steering committee members to go beyond the text to decode the meaning intended by the participants [13]. The thematic analysis process was adapted to include the following key stages: (1) studying the free-text suggestions to become familiar with the contributions made by the participants; (2) data coding to highlight key issues identified by the participants with regards to the definition and assessment of the dimensions; and (3) identifying patterns in the suggested modifications, developing themes, reflecting on these themes in the context of the overall data set, and defining the essence of each theme.

The themes were then used to revise the definitions and the assessment of the dimensions as appropriate. Feedback from the free-text suggestions and the changes that were made were also incorporated into the second round of the survey.

## Results

### Statistical Summary of Findings in the First Round

Thirty-five clinicians (including 26 doctors, 5 nurses, 2 pharmacists, 1 dietician, and 1 health system specialist) from 10 countries participated in the first round of this eDelphi study, with most being doctors (n=26, 74%) and male (n=23, 66%). About half of the participants had more than 10 years of digital health experience (n=18, 51%), and about half were from the United Kingdom (n=18, 51%). Most of the countries from which the participants came were high-income countries (8/10, 80%), although 1 of the 10 countries (10%) was lower middle income (Nigeria) and 1 (10%) was low income (the Gambia). Table 1 provides more detailed information on the sociodemographic characteristics of the participants.

In the first round of the eDelphi study, 86% to 97% of the clinicians ranked each of the 13 information quality dimensions in the proposed framework as relevant. These values were above the predefined threshold of 70% for the study and indicated consensus on the relevance of all 13 proposed dimensions in the framework. The ranking of the information quality dimensions is shown in Table 2.

**Table 1:** Sociodemographic characteristics of the eDelphi participants (N=35).

Characteristics		Participants, n (%)
**Occupation**		
	Doctor	26 (74)
	Nurse/nurse practitioner/advanced care practitioner	5 (14)
	Pharmacist/clinical pharmacist	2 (6)
	Dietician	1 (3)
	Health system specialist	1 (3)
**Digital health experience (years)**		
	Less than 10	17 (49)
	10 or more	18 (51)
**Country**		
	Croatia	1 (3)
	The Gambia	1 (3)
	Germany	1 (3)
	Ireland	5 (14)
	The Netherlands	3 (9)
	Nigeria	2 (6)
	Singapore	1 (3)
	United Arab Emirates	1 (3)
	United Kingdom	18 (51)
	United States of America	2 (6)
**Sex**		
	Male	23 (66)
	Female	12 (34)

**Table 2:** Ranking of the dimensions in the clinical information quality framework in the first round of the eDelphi study, with number of responses by participants (N =35) in selected categories.

Rank	Information quality dimension	“Strongly relevant,” n (%)	“Somewhat relevant,” n (%)	Combined relevance (“strongly relevant” or “somewhat relevant”), n (%)
1	Accuracy	30 (86)	2 (6)	32 (92)
2	Completeness	18 (51)	14 (40)	32 (91)
3	Interpretability	23 (66)	8 (23)	31 (89)
4	Plausibility	13 (37)	18 (51)	31 (89)
5	Provenance	27 (77)	7 (20)	34 (97)
6	Relevance	18 (51)	15 (43)	33 (94)
7	Accessibility	28 (80)	4 (11)	32 (91)
8	Portability	18 (51)	12 (34)	30 (86)
9	Security	25 (71)	5 (14)	30 (86)
10	Timeliness	25 (71)	9 (26)	34 (97)
11	Conformance	15 (43)	16 (46)	31 (89)
12	Consistency	10 (29)	20 (57)	30 (86)
13	Maintainability	20 (57)	14 (40)	34 (97)

### Changes Based on Free-Text Suggestions in the First Round

The changes that were made by the steering committee members based on the suggestions of the panel members in the first round are presented in Multimedia Appendix 2. The themes from the reflective thematic analysis of the free-text suggestions during the first round that informed these changes are presented in this section and summarized in Textbox 2.

**Themes from the free-text suggestions in the first round.**

- Avoiding ambiguity: this expresses the need to avoid ambiguous terms and phrases.
- Relatable examples: this indicates the recommendation to include examples relating to daily activities to make the questions and definitions more explicit.
- Renaming the dimensions: this relates to suggestions for naming and renaming of dimensions.
- Rephrasing for clarity: this expresses the need to rephrase aspects of the questionnaire to improve clarity.

#### Avoiding Ambiguity

The participants described some terms in the questionnaire as “vague,” “odd,” and “confusing.” For example, a participant stated the following about “errors”:

The term “errors” needs to be further defined, now it is too vague, and I have no idea what to think of when I read it.

In addition, some definitions were considered too complex to be understood by clinicians without informatics experience, as demonstrated by this comment:

Just at this point, I am thinking that it is relevant to understand who your audience is with these questions. Not all clinicians would understand these questions, but clinical informatics professionals would.

Several changes were made across the dimensions to avoid ambiguity, as recommended by the participants, including replacing or removing terms such as “free of errors,” “occasionally,” and “very” that were considered ambiguous by the participants.

#### Relatable Examples

Participants were unanimous that examples were useful in making questions more explicit. One participant advocated including an example for each option:

Give examples in each of the options, that would make it easier to differentiate.

On the other hand, another participant suggested including an example in the main question:

Perhaps include the example within the question, rather than the choice of answers.

Participants also advocated using specific examples that were relevant to daily activities of the clinicians. They proceeded to suggest examples they considered appropriate for each option.

Phone call to IT [information technology] dept is not sufficiently accessible, it’s another barrier (with a potential to fail- on hold, engaged, deadline, etc).

Pharma/tobacco or any other commercial marketing would be “very untrustworthy.”

However, participants acknowledged that it might be difficult to find suitable examples to illustrate some response options.

I’m struggling with the plausible/very plausible examples but can’t at this time think of an alternative.

Changes relating to this theme include introducing examples such as “two-factor authentication” to describe secure information and reassigning examples as suggested by participants, such as reassigning “access requiring phone call to IT [information technology] department” under “inaccessible” information.

#### Renaming Dimensions

Although all the dimensions were considered relevant, the free-text suggestions indicated a need for renaming some dimensions:

I don’t like the use of the word “interpretable” in the context of digital health records as it is too similar to “interoperable” and easily mis-read. Comprehensibility? Information clarity?

Some suggestions seemed to imply a need for a new dimension. A free-text suggestion on accessibility expressed concerns on how it might be difficult to search for information in a system holding the data.

I’d have the second option in the list, information is present in EHR [electronic health record] but have to spend time looking for it.

Multiple suggestions on “timeliness” seemed to indicate “currency” was favored over “timeliness.”

You could quickly log into a system that doesn’t contain the most up to date patient information which would be far more concerning in terms of data quality than logging in slowly to a system with the most recent info in it.

A new dimension, “searchability,” was introduced. In addition, “timeliness,” “provenance,” and “consistency” were renamed “currency,” “trustworthiness,” and “consistency of presentation,” respectively. Two suggestions from panel members that related to the renaming of dimensions but were not adopted to avoid ambiguity are presented in Multimedia Appendix 2.

#### Rephrasing for Clarity

Most of the suggested modifications related to the phrasing of the questionnaire. Each question and the associated options were rephrased as appropriate to clarify them. These modifications ranged from simple corrections such as typos to major changes introducing new ideas; these were addressed on a case-by-case basis.

The definition of an adverse event is too narrow. Consider reflecting both critical (patient safety) and non-critical (quality of care). Also, there is an implicit assumption that data will directly impact care - maybe use “contribute to” as opposed to “lead to.”

Thus, “adverse event” was replaced with an explanation of the likelihood that inaccurate information would affect quality of care and patient safety and the potential impact. Similarly, the phrase “intended task” was replaced with the term “patient care,” which is more all-encompassing. Other instances of rephrasing are presented in Multimedia Appendix 2.

### Results of the Second Round

A second round was conducted because the free-text suggestions indicated a need for an additional dimension. This round was also used to present the results of the first round to the participants and obtain further feedback on the modifications to the questionnaire. Full details on the modifications and point-by-point responses to the participants’ full-text suggestions for each of the dimensions are included in the questionnaire for the second round (Multimedia Appendix 3).

Among clinicians who provided their email addresses during the first round, 22 of 30 (73%) completed the second round. The threshold for consensus was reached for the new dimension “searchability.” Most of the participants agreed with the changes made to the definitions and assessments of the dimensions, ranging from 86% (n=19) for consistency of presentation to 100% (n=22) for accuracy, completeness, interpretability, maintainability, and searchability, with no further modifications suggested. Minor suggestions were made regarding rephrasing the definitions of plausibility, trustworthiness, accessibility, portability, security, conformance, and consistency of presentation. Multiple free-text suggestions indicated that the term “currency” was not as acceptable as “timeliness”:

I think timeliness and currency are two different terms that could not be used interchangeably. Therefore, I would prefer timeliness was not removed. if a result of an investigation is timely, it means it would be useful for decision making.

I don’t like the word currency in this context (it sounds like it’s referring to money).

The dimension “currency” was therefore reverted to the original name “timeliness.” The modified CLIQ framework is made up of 14 dimensions, as outlined in Table 3. The accompanying assessment questionnaire is presented in Multimedia Appendix 4.

**Table 3:** Clinical information quality framework for digital health.

Dimension		Description
**Informativeness (the usefulness of digital information for clinical purposes)**		
	Accuracy	The extent to which information is accurate.
	Completeness	The extent to which no required information is missing.
	Interpretability	The extent to which information can be interpreted.
	Plausibility	The extent to which information makes sense based on clinical knowledge.
	Trustworthiness	The extent to which the source of information is trustworthy and verifiable.
	Relevance	The extent to which information is useful for patient care.
**Availability (the functionality of the system holding clinical information)**		
	Accessibility	The extent to which information is accessible.
	Portability	The extent to which information can be moved or transferred between different systems.
	Searchability	The extent to which needed information can be found.
	Security	The extent to which information is protected from unauthorized access, corruption, and damage.
	Timeliness	The extent to which information is up-to-date.
**Usability (the ease of use of clinical information)**		
	Conformance	The extent to which information is presented in a format that complies with institutional, national, or international standards.
	Consistency of presentation	The extent to which presentation of information adheres to the same set of institutional, national, or international standards.
	Maintainability	The extent to which information can be maintained (eg, modified, corrected, updated, adapted, and upgraded) to achieve intended improvement.

## Discussion

### Principal Findings

This study was conducted to contextualize the CLIQ framework to the needs of clinicians. Consensus was reached on the relevance of all the existing dimensions and categories of the CLIQ framework, including informativeness (accuracy, completeness, interpretability, plausibility, provenance, and relevance), availability (accessibility, portability, security, and timeliness), and usability (conformance, consistency, and maintainability). A new dimension, searchability, was introduced in the “availability” category to account for the ease of finding needed information in the DHTs. “Provenance” and “consistency” were renamed “trustworthiness” and “consistency of presentation,” respectively.

The questionnaire was modified based on the suggestions of the clinicians to avoid ambiguities that could confuse users and affect the validity of the questionnaire. Nonspecific terms, such as “very,” “few,” or “occasionally,” were removed, as their meanings vary based on context. Certain dimensions, such as conformance, were redefined using nontechnical terms, making them comprehensible to clinicians without an informatics background. In addition, the clarity of the questionnaire was improved by rephrasing the questions, incorporating relatable examples, and renaming certain dimensions. Overall, these changes made the questionnaire more user-friendly and improved its face and content validity.

### Comparison With Prior Work

The CLIQ framework was developed to address gaps, including a lack of a pragmatic tool for clinical information quality assessment and the noninvolvement of clinicians in the development of existing frameworks [7]. The CLIQ framework is accompanied by a pragmatic questionnaire for assessing clinical information in DHTs, unlike theoretical frameworks, which provide no means of assessment [14-20]. The involvement of clinicians across 10 countries in the development of the CLIQ framework further differentiates the framework from existing frameworks, which were developed without input from clinicians [14,16-21]. Finally, the CLIQ framework is applicable to different DHTs, while existing frameworks are only applicable to specific DHTs, such as electronic health records [16,17,19,20,22].

### Strengths and Limitations

The eDelphi method afforded a systematic, practical, affordable, and transparent approach to integrating the opinions of multidisciplinary clinicians from 10 countries. The importance of multiple eDelphi rounds, which allow feedback on changes made in preceding rounds [9,10], was demonstrated in the rejection of the attempt to rename “timeliness” as “currency.” In addition, this study took advantage of the clinical experience and information governance expertise of the participating clinicians, thus combining practical user experience and subject matter expertise. The heterogeneous composition of the expert panel, which consisted of people from multiple clinical professions across 10 countries, enhanced the external validity of the CLIQ framework. However, external validity may be limited by the low proportion of participants from low- and middle-income countries. The snowball sampling technique might have contributed to the disproportionately higher number of participants who were doctors from the United Kingdom. Nevertheless, the participants in this study were actively engaged and went out of their way to scrutinize all the definitions and offer valuable suggestions to improve the CLIQ framework. Finally, the number of participants that completed the second round of the eDelphi study was modest (22/30, 73%) but this is still more than the 8 to 15 experts recommended in the literature for a Delphi study [8].

### Implications for Policy, Practice, and Future Research

This study provides insight into the information quality dimensions that are considered relevant by clinicians. Such insight could be useful when developing or choosing new DHTs for health care institutions. The consideration of relevant information quality dimensions while developing or choosing new DHTs will ensure that the information is fit for purpose. The CLIQ framework is thus a potential source of vital information to policy makers, DHT developers, and health care managers. In addition, the framework could be used to identify information quality problems in existing DHTs. As part of quality improvement projects, the CLIQ questionnaire could be used to collect data on the quality of information in existing DHTs from clinicians using these DHTs in clinical practice. Insight from such projects could then be used in planning strategies to address identified information quality problems.

The modification of the CLIQ framework has made the framework user-friendly by taking into account the views of the information users, as recommended in the information quality literature [23]. However, the adopted expert panel approach mainly improved the face and content validity of the framework [24]. Face and content validity imply that an instrument measures what it is intended to measure [24]. Therefore, a follow-up study to evaluate the construct validity and reliability of the CLIQ framework is ongoing across the United Kingdom among health care professionals who use the SystmOne electronic patient record system. Similar studies could be replicated in the future in low- and middle-income countries to further assess and, if needed, improve the applicability of the framework in such settings. The CLIQ framework will be made available under a Creative Commons (CC BY) license to facilitate its use in future works by other researchers who are interested in adapting the questionnaire based on their needs.

### Conclusions

The CLIQ framework reached a high expert consensus and clarity of language relating to the information quality dimensions. The study contextualized the questionnaire by obtaining direct input from clinicians who are users of clinical information in DHTs. The contextualized CLIQ framework offers a pragmatic approach to assessing clinical information in DHTs and could be used in practice to identify and forestall information quality problems that can compromise quality and safety of care.

## Appendix

Multimedia Appendix 1 First round eDelphi survey.

Multimedia Appendix 2 Changes based on free-text suggestions.

Multimedia Appendix 3 Second round eDelphi survey.

Multimedia Appendix 4 Clinical information quality (CLIQ) assessment questionnaire.

## Abbreviations

AI: artificial intelligence
CLIQ: clinical information quality
DHT: digital health technology

## Data Availability

Researchers can apply for access to the pseudonymized data by writing to the corresponding author.
