# Supplementary material for: Assessment of Clinical Information Quality in Digital Health Technologies: International eDelphi Study
Source: J Med Internet Res. 2022 Dec 6;24(12):e41889. doi: 10.2196/41889 (PMC9768639; doi:10.2196/41889)
Supplement: Multimedia Appendix 1 [file jmir_v24i12e41889_app1.docx]

**First Round eDelphi Survey**

**Clinical Information Quality Framework for Digital Health Technologies**

Digital health technologies (DHTs), such as electronic health records, clinical decision support systems and electronic prescribing systems, are widely used in patient care. Researchers at Imperial College London have developed an instrument for assessing the quality of clinical information from DHTs based on evidence from literature. This could help to prevent injuries and deaths associated with poor quality clinical information from DHTs. This study aims to obtain your inputs as a healthcare professional using information from DHTs. Kindly read further information about the study in the [participant information sheet](https://imperial.eu.qualtrics.com/CP/File.php?F=F_bQlDH1dgrZnlV9c).

If you are happy to proceed the with this study, please complete the consent form below:

I confirm that I have read and understood the participant information sheet, version 1.4 dated 3 November 2020, and have had the opportunity to ask questions which have been answered fully.

I give consent for information collected about me to be used to support other research in the future, including those outside of the European Economic Area (EEA).

I give consent to being contacted about the potential to take part in other research studies.

I understand that my participation is voluntary, and I am free to withdraw at any time, without giving any reason and without my legal rights being affected.

I give permission for Imperial College London to access my research records that are relevant to this research.

I consent to take part in the above study.

Please select which best describes your clinical role.

Doctor

Nurse/Nurse Practitioner/Advanced Care Practitioner

Pharmacist/ Clinical Pharmacist

Physiotherapist/Occupational Therapist

Physician Associate

Others (e.g., Community Health Worker, Healthcare Surveillance Officer)

If others, please specify

________________________________________________________________

In which country do you currently reside?

▼ Afghanistan ... Zimbabwe

Gender

Male

Female

Prefer not to say

Please state how long you have used digital health technologies such as electronic health record, electronic prescribing system, telemedicine and clinical decision support system in clinical practice?

1 year

2 years

3 years

4 years

5 years

6 years

7 years

8 years

9 years

10 years and above

How relevant to quality and safety of care do you consider each of these attributes of clinical information from DHTs?

|  | Strongly relevant | Somewhat relevant | Neither relevant nor irrelevant | Somewhat irrelevant | Strongly irrelevant |
| --- | --- | --- | --- | --- | --- |
| Accuracy: the extent to which information is free from errors. |  |  |  |  |  |
| Completeness: the extent to which no needed information is missing. |  |  |  |  |  |
| Interpretability: the extent to which information can be understood. |  |  |  |  |  |
| Plausibility: the extent to which information makes sense in the light of existing knowledge. |  |  |  |  |  |
| Provenance: the extent to which the source of information is trustworthy. |  |  |  |  |  |
| Relevance: The extent to which information is useful for the intended task. |  |  |  |  |  |
| Accessibility: the extent to which information is easily obtainable. |  |  |  |  |  |
| Portability: the extent to which information is available in different systems. |  |  |  |  |  |
| Security: the extent to which information is protected from unauthorized access. |  |  |  |  |  |
| Timeliness: the extent to which up-to-date information is available when needed. |  |  |  |  |  |
| Conformance: the extent to which information is presented in the desired format. |  |  |  |  |  |
| Consistency: the extent to which information is presented in the same format. |  |  |  |  |  |
| Maintainability: the extent to which information can be easily maintained. |  |  |  |  |  |

Would you include, exclude or modify the following question when assessing quality of clinical information from DHTs?

**Accuracy**: Is the information from the digital health technology free of errors?

☐ **Very Accurate**. The information from the digital health is completely free of errors.

☐ **Accurate**: The information from the digital health technology is free of errors that could lead to adverse events.

☐ **Inaccurate**: The information from the digital health technology contains few errors that could lead to adverse events.

☐ **Very inaccurate**: The information from the digital health technology contains several errors that could lead to adverse events. NB: Adverse event is an unintended physical injury resulting from or contributed to by medical care that requires additional monitoring, treatment or hospitalisation or that results in death.

Include as it is

Exclude

Modify

If modify, please state any modification you would like to suggest regarding definition and assessment of accuracy?

________________________________________________________________

________________________________________________________________

Would you include, exclude, or modify the following question when assessing quality of clinical information from DHTs?

**Completeness**: Is no needed information missing from the digital health technology?

☐**Very complete**: No information is missing from the digital health technology.

☐**Complete**: No information required for clinical decision (diagnosis, treatment or prognosis) is missing from the digital health technology.

☐**Incomplete**: Few information required for clinical decision (diagnosis, treatment or prognosis) are missing from the digital health technology.

☐**Very incomplete**: Several information required for clinical decision (diagnosis, treatment or prognosis) are missing from the digital health technology.

Include as it is

Exclude

Modify

If modify, please state any modification you would like to suggest regarding definition and assessment of completeness?

________________________________________________________________

________________________________________________________________

Would you include, exclude or modify the following question when assessing quality of clinical information from DHTs?
**Interpretability**: Could the information from the digital health technology be understood to make clinical decision?

☐**Very interpretable:** Additional resources provided to aid interpretation of the information from the digital health technology (e.g. arrows or colour coding to indicate abnormal results, indications of medication)

☐**Interpretable**: Standard resources provided to aid interpretation of the information from the digital health technology (e.g. reference range)

☐**Uninterpretable**: Information from the digital health technology cannot be interpreted without seeking clarification from its author.

☐**Very uninterpretable**: Completely meaningless information not suitable for clinical decision.

Include as it is

Exclude

Modify

If modify, please state any modification you would like to suggest regarding definition and assessment of interpretability?

________________________________________________________________

________________________________________________________________

Would you include, exclude or modify the following question when assessing quality of clinical information from DHTs?
**Plausibility**. Does the information from the digital health technology make sense based on common knowledge?

☐**Very plausible**. The information from the digital health technology agrees with common knowledge (e.g. raised inflammatory markers in a patient with sepsis).

☐**Plausible**. The information from the digital health technology agrees with common knowledge if exceptional circumstances are considered (e.g. normal inflammatory markers in a patient with sepsis due to delayed immune response)

☐**Implausible**: The information from the digital health technology disagrees with common knowledge (e.g. Arterial blood gasses with oxygen saturation of 60% when pulse oximeter records 94%)

☐**Very implausible**: The information from the digital health technology makes no sense at all based on common knowledge (e.g. physiological parameters incompatible with life).

Include as it is

Exclude

Modify

If modify, please state any modification you would like to suggest regarding definition and assessment of plausibility?

________________________________________________________________

________________________________________________________________

Would you include, exclude, or modify the following question when assessing quality of clinical information from DHTs?
**Provenance:** Is the source of the information in the digital health technology trustworthy? ☐**Very trustworthy**: The information in the digital health technology is from highly trustworthy source (e.g. UN Agencies, Official Government Agencies, Academic institutions, Hospitals).

☐**Trustworthy**: The information in the digital health technology is from recognised private corporations (e.g. non-governmental organisations, registered charities).

☐**Untrustworthy**: The information in the digital health technology is from sources with obvious conflict of interest (e.g. pharmaceutical companies, tobacco companies).

☐**Very untrustworthy**: Unverifiable source of information and unsubstantiated claims (e.g. broadcast information on social media, no references), unsuitable for clinical decision

Include as it is

Exclude

Modify

If modify, please state any modification you would like to suggest regarding definition and assessment of provenance?

________________________________________________________________

________________________________________________________________

Would you include, exclude, or modify the following question when assessing quality of clinical information from DHTs?

**Relevance:** Is the information from the digital health technology useful for the intended task? ☐**Very relevant**: All information from the digital health technology is useful for the intended task

☐**Relevant**: Most of the information from the digital health technology is useful for the intended task?

☐**Irrelevant**: Most of the information from the digital health technology not useful for the intended task

☐**Very irrelevant**: None of the information from the digital health technology is useful for the intended task

Include as it is

Exclude

Modify

If modify, please state any modification you would like to suggest regarding definition and assessment of relevance?

________________________________________________________________

________________________________________________________________

Would you include, exclude, or modify the following question when assessing quality of clinical information from DHTs?

**Accessibility:** Is the information easily obtainable from the digital health technology? ☐**Very accessible**: The information from the digital health technology is obtainable with no difficulties at the point of care.

☐**Accessible**: The information from the digital health technology is obtainable with minor difficulties that could be resolved at the point of care (e.g through a phone call to IT Department)

☐**Inaccessible**: The information from the digital health technology is not obtainable at the point of care.

☐**Very inaccessible**: The information from the digital health technology is not obtainable at all.

Include as it is

Exclude

Modify

If modify, please state any modification you would like to suggest regarding definition and assessment of provenance?

________________________________________________________________

________________________________________________________________

Would you include, exclude, or modify the following question when assessing quality of clinical information from DHTs?

**Portability:** Is the information from the digital health technology accessible in different systems?

☐**Very portable**: The information from the digital health technology is accessible at all levels of healthcare system (primary, secondary & tertiary).

☐**Portable**: The information from the digital health technology is accessible at all levels of healthcare with minor difficulties that could be resolved at the point of care (e.g. transferable on request).

☐**Unportable**: The information from the digital health technology is only accessible at the level of care where it was created.

☐**Very unportable**: The information from the digital health technology is only accessible on the computer system where it was created.

Include as it is

Exclude

Modify

If modify, please state any modification you would like to suggest regarding definition and assessment of provenance?

________________________________________________________________

________________________________________________________________

Would you include, exclude, or modify the following question when assessing quality of clinical information from DHTs?

**Security**: Is the information in the digital health technology protected from unauthorised access?

☐ **Very secure**: The information in the digital health technology is securely protected against unauthorized access using multiple strategies (e.g. password and swipe card).

☐ **Secure**: The information in the digital health technology is securely protected against unauthorised access using a single strategy (e.g. requires only password).

☐ **Insecure**: The information in the digital health technology is accessible to multiple healthcare professionals without a need for authorisation (e.g. information obtainable from the hospital without a need for personal log-in)

☐ **Very insecure**: The information is publicly accessible (e.g. information on hospital website)

Include as it is

Exclude

Modify

If modify, please state any modification you would like to suggest regarding definition and assessment of provenance?

______________________________________________________________

________________________________________________________________

Would you include, exclude, or modify the following question when assessing quality of clinical information from DHTs?

**Timeliness**: Is up-to-date information from the digital health technology available when it is needed?

☐ **Very timely**: Up-to-date information is available from the digital health technology at the point of care with no delays.

☐ **Timely**: Up-to-date information is available from the digital health technology at the point of care with minor delays which do not affect the use of the information for clinical decision (e.g. slow log-in)

☐ **Untimely**: Up-to-date information is unavailable from the digital health technology at the point of care due to major delays which affect the use of the information for clinical decision (e.g. system is down for a couple of hours)

☐ **Very untimely**: The information from the digital health technology is outdated and/or not available when needed for clinical decision

Include as it is

Exclude

Modify

If modify, please state any modification you would like to suggest regarding definition and assessment of provenance?

____________________________________________________________

________________________________________________________________

Would you include, exclude, or modify the following question when assessing quality of clinical information from DHTs?

**Conformance:** Is the information from the digital health technology presented in the desired format?

☐ **Very conformant**: All the information from the digital health technology conforms to international or local standards (e.g. SI units).

☐ **Conformant**: Most of the information from the digital health technology conforms to international or local standards.

☐ **Non-conformant**: Most of the information from the digital health technology do not conform to local or international standards.

☐ **Very conformant**: All the information from the digital health technology do not conform to local or international standards making it unsafe for clinical decision (e.g. medication doses presented without units).

Include as it is

Exclude

Modify

If modify, please state any modification you would like to suggest regarding definition and assessment of provenance?

________________________________________________________________

________________________________________________________________

Would you include, exclude, or modify the following question when assessing quality of clinical information from DHTs?

**Consistency**: Is the information presented in the same format within the digital health technology?

☐ **Very consistent**: All the information is presented consistently in the same format (e.g. consistently expressing Hb as g/dL) within the digital health technology.

☐ **Consistent**: Most of the information is presented consistently in the same format within the digital health technology.

☐ **Inconsistent**: Most of the information is not presented in the same format within the digital health technology.

☐ **Very inconsistent:** Multiple formats of information which is potentially confusing and unsafe for clinical decision.

Include as it is

Exclude

Modify

If modify, please state any modification you would like to suggest regarding definition and assessment of provenance?

________________________________________________________________

________________________________________________________________

Would you include, exclude, or modify the following question when assessing quality of clinical information from DHTs?

**Maintainability**: Could the information within the digital health technology be easily maintained?

☐ **Very maintainable**: The information within the digital health technology could be maintained without difficulties.

☐ **Maintainable**: The information within the digital health technology could be maintained with minor difficulties resolvable at the point of care.

☐ **Unmaintainable:** The information within the digital health technology could not be easily maintained.

☐ **Very maintainable**: The information within the digital health technology could not be maintained at all. NB: Maintainance includes activities such as storing, auditing, updating date.

Include as it is

Exclude

Modify

If modify, please state any modification you would like to suggest regarding definition and assessment of provenance?

________________________________________________________________

________________________________________________________________

Q35 CLIQ Framework for Digital Health Technology


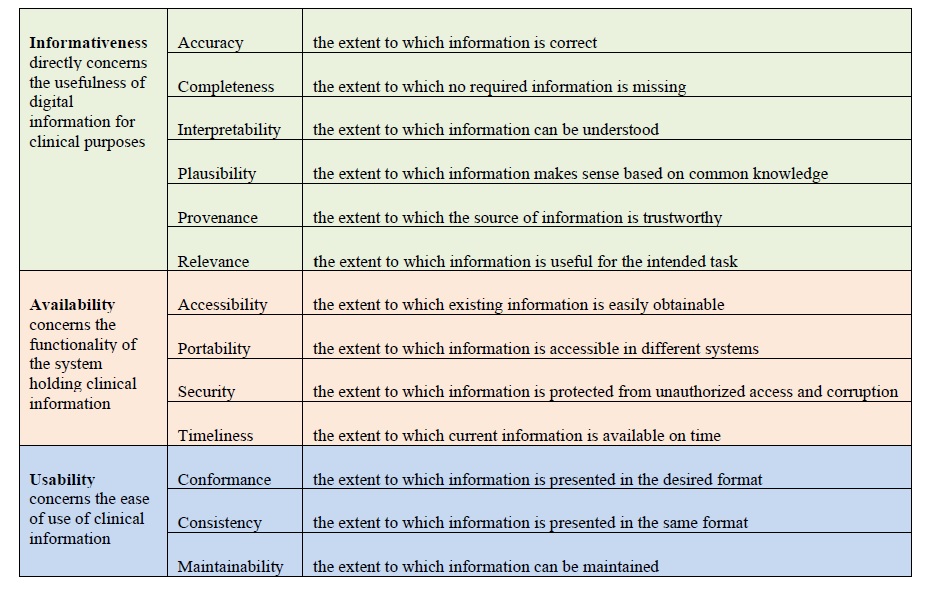


Q36 Would you like to retain or modify the above categories?

Retain the categories

Modify the categories

If modify, please state how you would want the categories to be modified.

________________________________________________________________

________________________________________________________________

________________________________________________________________

Thank you for taking part in this eDelphi survey. Please provide your email so we can share the summary of the findings with you and contact you for the subsequent round.

________________________________________________________________
