# Supplementary material for: Assessment of Clinical Information Quality in Digital Health Technologies: International eDelphi Study
Source: J Med Internet Res. 2022 Dec 6;24(12):e41889. doi: 10.2196/41889 (PMC9768639; doi:10.2196/41889)
Supplement: Multimedia Appendix 2 [file jmir_v24i12e41889_app2.docx]

**Changes based on free-text suggestions during the first round of the eDelphi study**

**Avoiding Ambiguity**

1. Removal of all response options that started with ‘very’ thus reducing the number of possible options to three (e.g., accurate, partially accurate, inaccurate).
2. The phrase ‘free of error’ was replaced with ‘accurate’ as the former was considered ambiguous.
3. The term ‘accessible’ was removed from the definition of portability to avoid confusion with ‘accessibility’.
4. Words indicating frequency (e.g., occasionally) and quantity (e.g., few) were removed as these could convey different meanings.
5. The phrase “partly interpretable” rather than the suggested “barely interpretable” was used to ensure uniformity with other dimensions.
6. ‘Conformance’ was defined using simpler language to make it comprehensible to clinicians without informatics experience.

**Relatable Examples**

1. ‘Transfer requiring printing on paper’ was included as an example of ‘unportable’ information
2. Diagnosis, monitoring, and treatment were included as examples of tasks under ‘relevance’.
3. ‘Access requiring phone call to IT department’ was re-assigned under ‘inaccessible’ information.
4. Two-factor authentication, smart card, and role-based access were included as examples of ‘secure’ information.
5. Examples for ‘timeliness’ were changed to reflect the validity of information in the system.
6. Examples of presentation format were included under ‘conformance’ and ‘consistency’.
7. Drop-down options and free text were included as examples of structured and unstructured information.
8. Community level of healthcare was included under ‘portability’.
9. Examples were included in each response option rather than in the question to make it easier to differentiate between options.

**Renaming Dimensions**

1. ‘Searchability’ was introduced to account for the ease of locating needed information in the DHTs.
2. ‘Timeliness’ was renamed as ‘currency’.
3. ‘Provenance’ was renamed as ‘trustworthiness’ to make it more comprehensible to clinicians without informatics background.
4. ‘Consistency’ was renamed ‘consistency of presentation’ to remove ambiguity.
5. ‘Interpretability’ was retained rather than the suggested ‘comprehensibility’ and ‘information clarity’ because healthcare professionals are likely to be more familiar with interpretation of information (e.g., lab results).
6. ‘Inconsistent’ was not used instead of ‘implausible’ as suggested because it might create confusion with ‘consistency’.
7. ‘Portability’ was not changed to ‘interoperability’ as suggested because ‘portability’ describes an attribute of information while ‘interoperability’ describes the attribute of a digital system.

**Rephrasing for Clarity**

1. *‘*Adverse event’ was replaced with explanation indicating likelihood and potential impact of inaccurate information on quality of care and patient safety.
2. Options for accuracy were rephrased to remove the implicit assumption that data will directly impact care.
3. ‘Is no information missing?’ was rephrased to ‘Is any information missing?’
4. ‘Clinical decision’ was changed to ‘decision making’ which is more encompassing.
5. ‘Intended task’ was replaced with ‘patient care’ as the latter is all-encompassing.
6. Definitions of ‘maintainability’ and ‘security’ were rephrased to make them more explicit.
7. Transfer between different systems in addition to different levels of healthcare were included in the definition of ‘portability’.
8. ‘Healthcare institutions’ was used instead of suggested ‘NHS Trust’ as the latter is specific to the UK.
