# Supplementary material for: Assessment of Clinical Information Quality in Digital Health Technologies: International eDelphi Study
Source: J Med Internet Res. 2022 Dec 6;24(12):e41889. doi: 10.2196/41889 (PMC9768639; doi:10.2196/41889)
Supplement: Multimedia Appendix 3 [file jmir_v24i12e41889_app3.docx]

**Second Round eDelphi Survey**

**Clinical Information Quality Framework for Digital Health Technologies**

The aim of this study is to obtain inputs of clinicians on an instrument for assessing the quality of clinical information from digital health technologies. Thirty-five healthcare professionals (26 doctors, 5 nurses, 2 pharmacists, 1 dietician, 1 health system specialist) from 10 countries participated in the first round with more than half (51%) having over ten years digital health experience. All the 13 information quality dimensions in the proposed framework were ranked as relevant by the majority of the first-round participants with scores ranging from 86% to 94%. This final round seeks to obtain your feedback on the modifications that have been made. Please read the [participant information sheet](https://imperial.eu.qualtrics.com/CP/File.php?F=F_bQlDH1dgrZnlV9c) for further information about the study.

**General Comments (Applicable to all dimensions)**

1. We are grateful for all the comments. They are very useful in providing further insights into the assessment of each dimension.
2. Based on the comments, we have reduced the options from 4 to 3. We have removed the ‘very’ option as this might create some ambiguity.
3. We have also removed the phrase ‘digital health technology’ which was used repeatedly during the first survey. We believe that using this term once at the introduction to the instrument will suffice.
4. We have removed timeliness and introduced 2 new dimensions: currency and searchability based on comments during the first survey.
5. We have responded to the comments made during the first round one by one.
6. The responses are based on the discussion among the steering committee members for the eDelphi study made up of seven digital health researchers from Imperial College London.
7. We would appreciate further comments on each dimension.

**Accuracy: Is the information accurate?**
☐ **Accurate**. The information is accurate.
☐ **Partly accurate**: The digital health technology contains some inaccurate information which is unlikely to compromise patient safety or quality of care (i.e., unlikely to cause death, harms or disruption/delay in patient care). 
☐ **Inaccurate**: The digital health technology contains some inaccurate information which is likely to compromise patient safety or quality of care (i.e., likely to cause death, harms or disruption/delay in patient care).

**Response to comments during the first round of the survey**

We acknowledge that the term “free of error” could be ambiguous and have replaced it with the term “accurate”

We agree that the definition of adverse event is narrow and have replaced it with likelihood and potential impact of inaccurate information on quality of care and patient safety.

We agree that adverse events and accuracy are not mutually exclusive. This is reflected in the ‘partly accurate’ and ‘inaccurate’. Similarly, we have used the term ‘likely’ and ‘unlikely’ to remove the implicit assumption that data will directly impact care.

We did not add the phrase ‘to the best of my knowledge’ to this question because this is applicable to all questions as responses are always based on the knowledge and experience of respondents to any questionnaire. We will make this clear in the introductory statement to the questionnaire.

We have not listed different types of errors (e.g., medication, history findings, etc.) because these differ across different digital health technologies and it is impracticable to list all of them.

We acknowledge that data input has an impact on information quality. We hope that this instrument will help with discovery of poor quality information which will in-turn help the relevant authorities to look closely at the possible causes including data entry error.

**Please indicate whether you agree with these changes.**

- Yes
- No

**Please state any further modification you would like to suggest regarding the definition and assessment of accuracy**.

________________________________________________________________

________________________________________________________________

________________________________________________________________

**Completeness: Is any information missing?**
 ☐ **Complete**: No information reasonably expected to be documented/recorded is missing (e.g., name, dose and indication of medication prescribed).
 ☐ **Partly complete**: Information reasonably expected to be documented/recorded is partially recorded (e.g., name of medication recorded without the dose).
 ☐ **Absent**: Information reasonably expected to be documented is absent/missing (e.g., name, dose and indication of medication missing).

**Response to comments during the first round of the survey**

We have replaced “no” with “any” in the question as suggested.

We agree that the function of digital health technology is beyond clinical decision making, we have therefore removed this phrase.

We have however refrained from using words indicating frequency (e.g., occasionally, usually) and quantity (e.g., few) to avoid ambiguity as these could have different meanings for different people.

**Please indicate whether you agree with these changes.**

- Yes
- No

Please state any further modification you would like to suggest regarding the definition and assessment of completeness

________________________________________________________________

________________________________________________________________

________________________________________________________________

**Interpretability: Can the information be interpreted?**
 ☐ **Interpretable**: The information can be interpreted without a need for additional resources (e.g., reference range included alongside laboratory results).
 ☐ **Partly interpretable**: The information can be interpreted only with additional resources or assistance (e.g., reference materials required because reference range not included with laboratory results)
 ☐ **Uninterpretable**: The information cannot be interpreted even with additional resources (e.g., unable to interpret laboratory results even after consulting reference materials).

**Response to comments during the first round of the survey**:

We have modified the options to demonstrate some sort of order across the interpretability dimension as suggested.

We acknowledge that the phrases very interpretable and very uninterpretable sound odd, we have therefore removed these terms.

We have used the phrase “partly interpretable” rather than “barely interpretable” suggested to ensure uniformity with other dimensions.

Although we acknowledge that the term “comprehensibility” and “information clarity” are similar, we have retained “interpretability” because we believe healthcare professionals are more familiar with interpretation of information (e.g., lab results) compared with other suggested alternatives.

**Please indicate whether you agree with these changes?**

- Yes
- No

Please state any further modification you would like to suggest regarding definition and assessment of interpretability.

________________________________________________________________

________________________________________________________________

________________________________________________________________

**Plausibility. Does the information seem credible, reasonable, or likely?**
 ☐ **Plausible**. The information seems credible, reasonable, or likely/probable (e.g., fever in a child with sepsis).
 ☐ **Partly plausible**. The information seems probable only in exceptional circumstances (e.g., normal body temperature in a child with sepsis)
 ☐ **Implausible**: The information is unlikely to be true (e.g., body temperature of 100 degrees Celsius which is not compatible with life)

**Response to comments during the first round of the survey**

We have modified the examples and relate them to a single scenario (sepsis) as suggested.

Although we considered using the term “consistent with common knowledge”, we avoided this phrase because it might create confusion with another dimension (consistency).

**Please indicate whether you agree with these changes.**

- Yes
- No

Please state any further modification you would like to suggest regarding definition and assessment of plausibility.

________________________________________________________________

________________________________________________________________

________________________________________________________________

**Trustworthiness**: Is the source of the information trustworthy and verifiable? 
☐ **Trustworthy**: The information is from a trustworthy source (e.g., WHO, government agencies, academic institutions, healthcare institutions, peer-reviewed medical journals). 
☐ **Partly trustworthy**: The information is only partly from a verifiable source (e.g., some claims are without evidence-base). 
☐ **Untrustworthy**: The information is from unverifiable and non-peer reviewed sources (e.g., social media).

**Response to comments during the first round of the survey**

We have replaced the term “provenance” with trustworthiness as the latter is easier to understand by healthcare professionals without informatics background.

We have used the term healthcare institutions and avoided the term “NHS Trust” which is specific to the UK.

We have retained multiple examples to make the classification more apparent.

**Please indicate whether you agree with these changes**.

- Yes
- No

Please state any further modification you would like to suggest regarding definition and assessment of trustworthiness.

________________________________________________________________

________________________________________________________________

________________________________________________________________

**Relevance: Is the information relevant to patient care?**
☐ Relevant: The information is useful, directly connected with or related to patient care (e.g., useful for diagnosis, monitoring and treatment).
 ☐ Partly relevant: The information is useful, directly connected with or related to some aspects of patient care (e.g., useful only for discharge planning).
 ☐ Irrelevant: The information is not useful, directly connected with or related to any aspect of patient care.

 **Response to comments during the first round of the survey**:

We have replaced the term ‘intended task’ with ‘patient care’ as the latter is all-encompassing.

We have included diagnosis, monitoring and treatment as examples of patient care as suggested.

**Please indicate whether you agree with these changes**

- Yes
- No

Please state any further modification you would like to suggest regarding definition and assessment of relevance.

________________________________________________________________

________________________________________________________________

________________________________________________________________

**Accessibility: Can the information be obtained or reached easily?** 
☐ **Accessible**: The information can be easily obtained at the point of care (e.g., immediate access with username and password). 
☐ **Partly accessible**: The information can be obtained with some difficulties (e.g., delayed access due to frequent downtime).
 ☐ **Inaccessible**: The information is not easily obtainable (e.g., access requires phone calls to IT Department).

**Response to comments during the first round of the survey**:

We have included examples in each option rather than in the question as we agree with the suggestion that this would make it easier to differentiate.

We agree with the comments that “phone call to IT department” is an example of inaccessible information.

**Please indicate whether you agree with these changes**.

- Yes
- No

Q16 Please state any further modification you would like to suggest regarding definition and assessment of accessibility.

________________________________________________________________

________________________________________________________________

________________________________________________________________

**Portability**: Is the information easily moved or transferred? Portability is a feature of information that allows a user to take information from one system or part of the system and transfer or “port” it to another part of the system or elsewhere (e.g., community, primary, secondary, tertiary care). 
☐ **Portable**: The information is easily moved or transferred (e.g., transferable at the point of care by clinician). 
☐ **Partly portable**: The information is transferable partly or transfer requires additional effort (e.g., conversion of values). 
☐ **Unportable**: The information is not transferable to other system or other part of the system (e.g., requires printing). 
 
**Response to comments during the first round of the survey**:

We have removed the term “accessible” from the definition to avoid confusion with accessibility dimension.

We have included transfer between different systems in addition to levels of healthcare.

We have included the community level of healthcare.

We have included printing as an example of unportable.

We have used the term portability because it describes an attribute of information rather than interoperability which describes an attribute of the digital system. The primary purpose of this instrument is to assess clinical information rather than the digital system.

**Please indicate whether you agree with these changes**

- Yes
- No

Please state any further modification you would like to suggest regarding definition and assessment of portability.

________________________________________________________________

________________________________________________________________

________________________________________________________________

**Security: Is the information protected from unauthorized access, corruption, damage and loss throughout its lifecycle across all applications and platforms?**
☐ **Secure**: The information is protected from unauthorized access, corruption, damage and loss using a comprehensive strategy (this includes but not limited to two-factor authentication, password and smart card, role-based access, encryption, single-sign-on authentication). 
☐ **Partly secure**: The information is protected from unauthorized access, corruption, damage and loss by only some rather than comprehensive strategy (e.g., requires only username and password but not role-based access). 
☐ **Insecure**: The information is not protected from unauthorized access, corruption and loss (e.g., information obtainable from the hospital system using a generic account).
 
**Response to comments during the first round of the survey**

We have included different examples suggested such as two-factor authentication, smart card, role-based access etc.

We have added the security level that are deemed safe under the “secure” option.

We acknowledge that security is quite broad but we have tried to simplify it so that it could be understood by healthcare professionals without informatics expertise.

**Please indicate whether you agree with these changes**

- Yes
- No

Please state any further modification you would like to suggest regarding definition and assessment of security.

________________________________________________________________

________________________________________________________________

________________________________________________________________

**Conformance**: Is the information presented in a format that complies with national standards/guidelines? 
Note: Presentation format includes but not limited to units (e.g., Celsius, Fahrenheit), structure (e.g., drop down, free text), scoring system (e.g., GCS- Glasgow Comma Scale). abbreviation (Hb vs Hemoglobin).
☐ **Conformant**: the information complies with national standards/guidelines (e.g., presentation of level of consciousness using GCS stating all the components). 
☐ **Partly conformant**: the information partly complies with national standards/guidelines (e.g., presentation of level of consciousness using GCS stating only the total score). 
☐ **Non-conformant**: the information does not comply with national standards/guidelines (e.g., presentation of level of consciousness not using GCS or any other recommended scoring system). 
 
**Response to comments during the first round of the survey**

We have tried to define the term conformance using simpler language and common international example to illustrate it.

We have included examples of presentation format to make the definition more explicit.

We have included drop down and free text as an example of structured and unstructured information.

**Please indicate whether you agree with these changes.**

- Yes
- No

Please state any further modification you would like to suggest regarding definition and assessment of conformance.

________________________________________________________________

________________________________________________________________

________________________________________________________________

**Consistency of presentation**. Does the presentation of information adhere to the same set of principles / consistently in the same format? 
Note: Presentation format includes but not limited to units (e.g., Celsius, Fahrenheit), structure (e.g., drop down, free text), scoring system (e.g., GCS- Glasgow Comma Scale). abbreviation (Hb vs Hemoglobin)
☐ **Consistently presented**: The information is presented consistently in the same format (e.g., Adrenaline dose consistently expressed in mg across the system). 
☐ **Consistently presented partly**: The information is presented in multiple formats in different parts of the system (Adrenalin dose presented as mg and ml in different parts of the system). 
☐ **Inconsistently presented**: The information is presented in multiple formats in the same part of the system (Adrenalin dose presented as mg and ml within the same drop-down). 
 
**Response to comments during the first round of the survey**

1. We have used examples to illustrate each option
2. We have included examples of presentation format to make the definition more explicit.

**Please indicate whether you agree with these changes.**

- Yes
- No

Please state any further modification you would like to suggest regarding definition and assessment of consistency of presentation.

________________________________________________________________

________________________________________________________________

________________________________________________________________

**Maintainability**: Can the information be easily modified to achieve intended improvement? 
Note: Maintainability includes a range of activities such as correction, updating, upgrading, adaptation carried out by healthcare professionals or support staff
☐ **Maintainable**: The information can be modified without any difficulties (e.g., immediate correction of wrong entry in electronic health records automatically approved with audit trail). 
☐**Partly maintainable**: The information can be modified with resolvable difficulties (e.g., correction of wrong entry in the electronic health records requires authorization by another professional who is not its author). 
☐ **Unmaintainable:** The information cannot be modified (e.g., wrong entry cannot be corrected but requires additional entry such as an addendum to explain the modifications). 
 
**Response to comments during the first round of the survey**

1. We have rephrased the definition of maintainability and added a note to make it clearer.
2. We have provided relatable examples.

**Please indicate whether you agree with these changes.**

- Yes
- No

Please state any further modification you would like to suggest regarding definition and assessment of maintainability.

________________________________________________________________

________________________________________________________________

________________________________________________________________

**Currency: Is the information current?**
☐ **Current**: The information is current (e.g., current laboratory results available). 
☐ **Partly current**: The available information is not current, but useful for patient care (e.g., historical laboratory results showing trends of chronic disease management). 
☐ **Non-current**: The information is outdated (e. g., current laboratory results not in the records of an acutely ill person).
 
**Response to comments during the first round of the survey**

1. We have changed the term ‘timeliness’ to ‘currency’ because we believe that the other aspect of timeliness relating to being available when needed is already captured under accessibility dimension.
2. We have changed the examples to reflect the validity of information in the system rather than the accessibility aspect.

**How relevant to quality and safety of care do you consider currency of information in the digital health technology?**

- Strongly relevant
- Somewhat relevant
- Neither relevant nor irrelevant
- Somewhat irrelevant
- Strongly irrelevant

Please state any comments/modification you would like to suggest regarding definition and assessment of currency.

________________________________________________________________

________________________________________________________________

________________________________________________________________

**Searchability: Can the information be easily searched for or retrieved?**
☐ **Searchable**: The information is easily searched for or retrieved at the point of care.
☐ **Partly Searchable**: The information can be searched for or retrieved at the point of care with some difficulty.
☐ **Not Searchable**: The information cannot be easily searched for or retrieved at the paoint of care.
 
**Comment:**This is a new dimension introduced based on comments on accessibility and timeliness during the first round of survey.

**How relevant to quality and safety of care do you consider searchability of information in the digital health technology?**

- Strongly relevant
- Somewhat relevant
- Neither relevant nor irrelevant
- Somewhat irrelevant
- Strongly irrelevant

Please state any comments/ modification you would like to suggest regarding definition and assessment of searchability

________________________________________________________________

________________________________________________________________

________________________________________________________________

Thank you for taking part in the survey. Please provide your email if you would like to be included as co-author/contributor of the manuscript.
